# Does the Blood–Brain Barrier Integrity Change in Regard to the Onset of Fetal Growth Restriction?

**DOI:** 10.3390/ijms24031965

**Published:** 2023-01-19

**Authors:** Natalia Misan, Sławomir Michalak, Katarzyna Kapska, Krystyna Osztynowicz, Mariola Ropacka-Lesiak, Katarzyna Kawka-Paciorkowska

**Affiliations:** 1 Department of Perinatology and Gynecology, Poznan University of Medical Sciences, 60-535 Poznan, Poland; 2 Department of Neurochemistry and Neuropathology, Poznan University of Medical Sciences, 60-355 Poznan, Poland; 3 Department of Neurosurgery and Neurotraumatology, Poznan University of Medical Sciences, 60-355 Poznan, Poland

**Keywords:** early-onset fetal growth restriction, late-onset fetal growth restriction, placental insufficiency, blood–brain barrier, fetal hypoxia, brain damage, tight junctions, tight junction proteins

## Abstract

The aim of the study was to determine whether early-onset and late-onset fetal growth restriction (FGR) differentially affects the blood–brain barrier integrity. Furthermore, the purpose of the study was to investigate the relationship between the blood–brain barrier breakdown and neurological disorders in FGR newborns. To evaluate the serum tight junction (TJ) proteins and the placental TJ proteins expression, an ELISA method was used. A significant difference in serum OCLN concentrations was noticed in pregnancies complicated by the early-onset FGR, in relation to the intraventricular hemorrhage (IVH) occurrence in newborns. No significant differences in concentrations of the NR1 subunit of the *N*-methyl-d-aspartate receptor (NR1), nucleoside diphosphate kinase A (NME1), S100 calcium-binding protein B (S100B), occludin (OCLN), claudin-5 (CLN5), zonula occludens-1 (zo-1), the CLN5/zo-1 ratio, and the placental expression of OCLN, CLN5, claudin-4 (CLN4), zo-1 were noticed between groups. The early-onset FGR was associated with a higher release of NME1 into the maternal circulation in relation to the brain-sparing effect and premature delivery. Additionally, in late-onset FGR, the higher release of the S100B into the maternal serum in regard to fetal distress was observed. Furthermore, there was a higher release of zo-1 into the maternal circulation in relation to newborns’ moderate acidosis in late-onset FGR. Blood–brain barrier disintegration is not dependent on pregnancy advancement at the time of FGR diagnosis. NME1 may serve as a biomarker useful in the prediction of fetal circulatory centralization and extremely low birth weight in pregnancies complicated by the early-onset FGR. Moreover, the serum zo-1 concentration may have prognostic value for moderate neonatal acidosis in late-onset FGR pregnancies.

## 1. Introduction

Early-onset FGR, which is diagnosed up to 32 weeks of gestation, affects about 1–2% of pregnant women and accounts for 20–30% of all FGR cases [1,2]. It is associated with severe placental insufficiency and abnormalities in Doppler ultrasound [3,4,5], which leads to systemic adaptation in the circulatory system, with subsequent decompensated hypoxia and acidosis [6]. Although fetal immaturity increases its tolerance to hypoxia [7], high morbidity and mortality rates are observed [8]. A crucial issue is the proper management and monitoring of pregnancy to assess the benefits of conservative management, the risk of increased fetal adverse perinatal outcomes, as well as the consequences of prematurity [1]. Late-onset FGR, defined as growth retardation evolved at or above 32 gestational weeks, complicates about 3–5% of pregnancies, accounting for 70–80% of all FGR cases [1,9,10]. It co-occurs with a mild placental insufficiency, although the cerebroplacental ratio may be decreased [11,12]. As fetal maturity is reached, its tolerance to hypoxia is reduced, which leads to circulatory centralization (brain-sparing effect) [1,13]. Neonatal mortality is lower, while late-onset intrauterine fetal death (IUFD) is found more frequently. Late-onset FGR is associated with the risk of significant fetal deterioration and neonatal acidosis. Moreover, sudden fetal decompensation may occur without previously observed symptoms, leading to neurodevelopmental abnormalities or IUFD [1,14,15]. The main challenge in late-onset FGR is the appropriate diagnosis to prevent neurological deficits and late-onset IUFD [1,16]. Importantly, previous studies have revealed that none of the Doppler findings showed a significant relationship with the subsequent development of IVH in newborns [17].

Disrupted blood flow leads to cerebral ischemia, which is associated with the rapid depletion of essential nutrients and oxygen [18]. Placental insufficiency has neuropathological consequences for fetal neurodevelopment, and the spectrum of these abnormalities is diversified [19,20]. Critical parameters for fetal brain injury are at the time of placental failure and the gestational age at delivery [21,22]. Both the early-onset and late-onset FGR are significant risk factors for cerebral palsy [22,23,24], however, it is currently unknown whether the early- and late-onset FGR differentially affect the blood–brain barrier permeability.

The blood–brain barrier is a semipermeable border of endothelial cells, whose function is the protection of the nervous system from harmful agents as well as ensuring the selective transport of substances from the blood into the cerebrospinal fluid [25]. It is composed of microvascular endothelial cells, which line the cerebral capillaries and penetrate most organisms’ brain and spinal cord with a well-developed central nervous system [26]. The brain microvascular endothelial cells characterize unique morphology, structure, and function that vary them from other blood vessels. They exhibit the expression of TJ proteins, which seal the paracellular permeability for unregulated passage of molecules between blood and brain [27]. The TJ are formed by integral membrane proteins, such as claudins, OCLN, and junctional adhesion molecules (JAMs), which are linked to the actin cytoskeleton by cytoplasmic proteins, such as zonula occludens-1, -2, and -3 protein (zo-1, zo-2, and zo-3), cingulin, and others [28,29]. The changes in the blood–brain barrier permeability are possible to observe in vivo in ischemic stroke patients [30,31,32], and just the reports about the effects of hypoxia on the blood–brain barrier breakdown in patients with neurological disorders have become the basis for drawing hypotheses of our study.

The aim of the study was to assess the damage to the endothelial TJ in pregnancies complicated by the early-onset and late-onset FGR, leading to the appearance of the TJ proteins (OCLN, CLN5, and zo-1) and molecules indicative of neuronal damage (NR1, NME1, S100B) in maternal blood. Moreover, the objective was to determine whether the early-onset and late-onset FGR affect the blood–brain barrier integrity in a different way. Furthermore, the purpose of the study was to evaluate the relationship between the blood–brain barrier breakdown and perinatal outcomes, especially neurological disorders in newborns, as well as the possibility of their prediction by the use of studied biochemical parameters. In addition, the expression of the placental TJ proteins (OCLN, CLN4, CLN5, and zo-1) were also evaluated.

## 2. Results

### 2.1. Characteristics of Early-Onset and Late-Onset FGR Group

The early-onset and late-onset FGR were diagnosed at an average 30 and 36 weeks of gestation, respectively. The groups were age, BMI, and gestational weight gain-matched. The women did not differ according to gravidity and parity (Table 1). The average estimated fetal weight in ultrasound was 1265 g and 2211 g in the early-onset and late-onset FGR groups, accordingly. The significantly higher umbilical artery pulsatility index, left uterine artery pulsatility index, uterine artery score, frequency of the abnormal umbilical artery pulsatility index, umbilical artery absent end-diastolic flow, abnormal left and right uterine artery pulsatility index, and abnormal ductus venosus pulsatility index were noticed in the early-onset FGR as compared to the late-onset FGR group. Moreover, the lower cerebroplacental ratio and the higher occurrence of the brain-sparing effect was observed significantly more often in the early growth-restricted fetuses at a less advanced gestational week in comparison to the late-onset FGR (Table 2).

### 2.2. Serum Biochemical Measurements

No significant differences in the concentrations of NR1, NME1, S100B, OCLN, CLN5, and zo-1 and the values of CLN5/zo-1 ratio were observed between the early-onset and late-onset FGR groups (Table 3).

### 2.3. Placental TJ Proteins Expression

No statistical differences in placental expression of OCLN, CLN5, CLN4, and zo-1 were noticed between groups (Table 4).

### 2.4. Perinatal Outcomes of Growth-Restricted Fetuses

The IUFD occurred in 5.1% of the early-onset FGR pregnancies, whereas it was not observed in late-onset FGR. The women with the early-onset FGR delivered significantly earlier, and as a result, the rate of preterm labor was significantly higher among them. No differences in the mode of delivery were noticed between groups. Fetal distress was observed significantly more often in the early-onset FGR group. No differences in perilabor blood loss and placental weight were noticed between groups (Table 5). The early-onset FGR newborns had significantly lower birth weight with a higher incidence of very low and extremely low ones. The 1-min and 5-min Apgar scores were significantly lower in the early-onset FGR newborns. The venous pH and BE, arterial pH and BE, as well as the frequency of acidosis were comparable between groups. Respiratory distress syndrome and respiratory failure were significantly more common in the early-onset FGR. The frequency of bronchopulmonary dysplasia, congenital pneumonia, necrotizing enterocolitis, retinopathy, and neonatal death during hospitalization was comparable between groups (Table 6).

### 2.5. Assessment of Biochemical Parameters versus Neurological Complications

**Figure 1:**
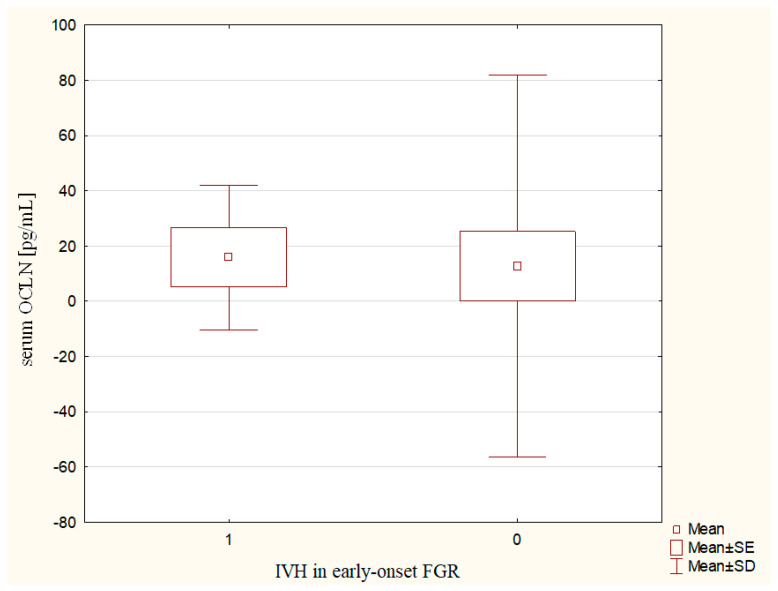
The maternal serum OCLN concentrations in relation to newborns IVH in the early-onset FGR.

The IVH was significantly more common in the early-onset FGR group, while only one case was confirmed in the late-onset FGR. The frequency of periventricular leukomalacia did not differ statistically between groups but it was not noticed in the late-onset FGR (Table 6). A significant difference in serum OCLN concentrations was observed in pregnancies complicated by the early-onset FGR, in relation to the IVH occurrence in newborns. Mothers from the early-onset FGR group, whose newborns developed IVH after delivery, had significantly higher serum OCLN concentrations (15.83 ± 26.19 vs. 12.63 ± 69.20 pg/mL, p ~ 0.0239) as compared to pregnancies with the early-onset FGR without postpartum IVH (Figure 1).

### 2.6. Setting Up a Role of Studied Proteins as Biomarkers in the Prediction of Adverse Perinatal Outcomes

**Figure 2:**
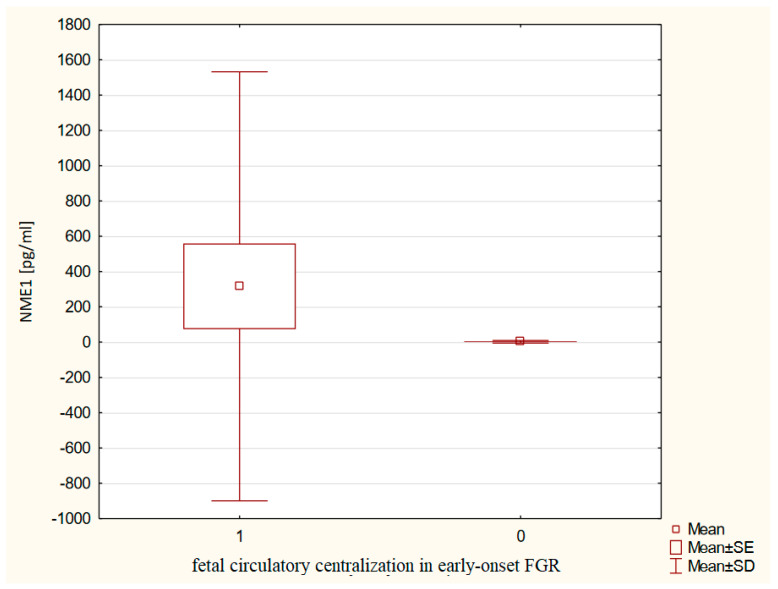
The maternal serum NME1 concentrations in relation to fetal circulatory centralization in the early-onset FGR.

**Figure 3:**
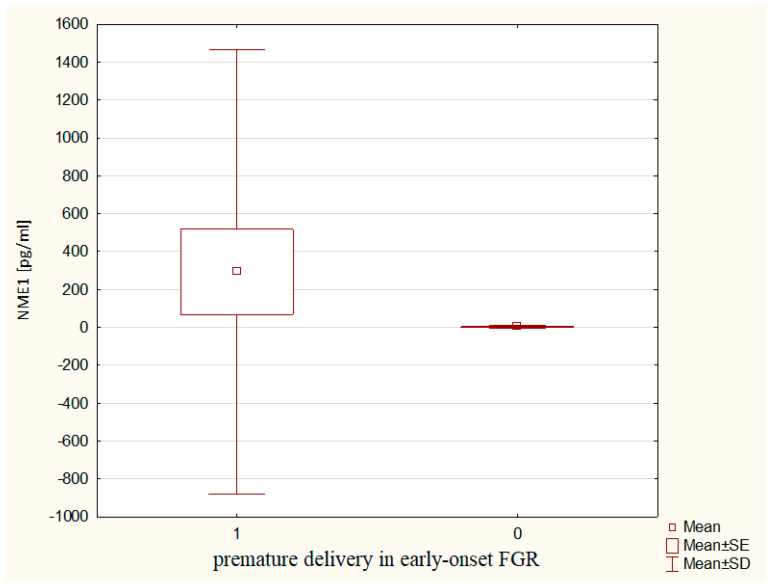
The maternal serum NME1 concentrations in relation to premature delivery in the early-onset FGR.

A significant relationship between serum NME1 concentrations and the brain-sparing effect was found in pregnancies complicated by the early-onset FGR. Mothers from the early-onset FGR group with fetal brain-sparing effect had significantly higher serum NME1 levels (316.16 ± 1215.52 vs. 2.02 ± 7.30 pg/mL, *p* ~ 0.0098) as compared to those with the early-onset FGR without circulatory centralization (Figure 2). Furthermore, the women in pregnancies complicated by the early-onset FGR, who delivered prematurely, had significantly higher NME1 concentrations (292.70 ± 1170.87 vs. 2.63 ± 8.32 pg/mL, *p* ~ 0.0370) in comparison to mothers with the early-onset FGR, who delivered at term (Figure 3). The role of serum NME1 concentration in the prediction of the circulatory centralization in the early-onset FGR was significant (cut-off value for NME1: 26.31 pg/mL, sensitivity 0.48, specificity 0.92, PPV 0.92, NPV 0.48, AUC 0.720, *p* ~ 0.0278). Moreover, the usefulness of serum NME1 concentration in the prognosis of extremely low birth weight was proven (cut-off value for NME1: 38.97 pg/mL, sensitivity 0.62, specificity 0.88, PPV 0.73, NPV 0.81, AUC 0.723, *p* ~ 0.0270) in the early-onset FGR group.

**Figure 4:**
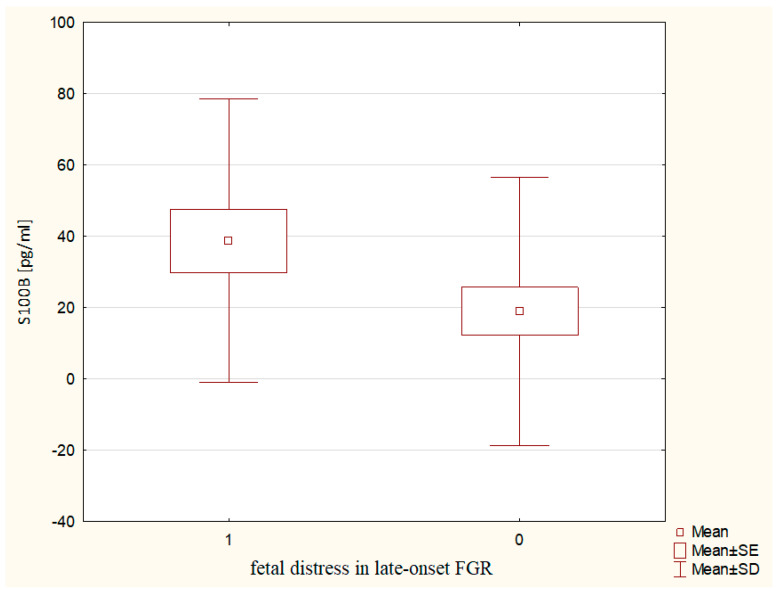
The maternal serum S100B concentrations in relation to fetal distress in the late-onset FGR.

A significant difference between the S100B concentrations was observed in pregnancy complicated by the late-onset FGR, according to the occurrence of fetal distress. Mothers from the late-onset FGR group, who developed perinatal fetal life-threatening symptoms, had significantly higher S100B levels (38.80 ± 39.77 vs. 19.00 ± 37.68 pg/mL, p ~ 0.0309) as compared to pregnancies without fetal distress (Figure 4). Despite the different serum S100B concentrations, no predictive role for this protein in the prognosis of fetal distress was noticed.

**Figure 5:**
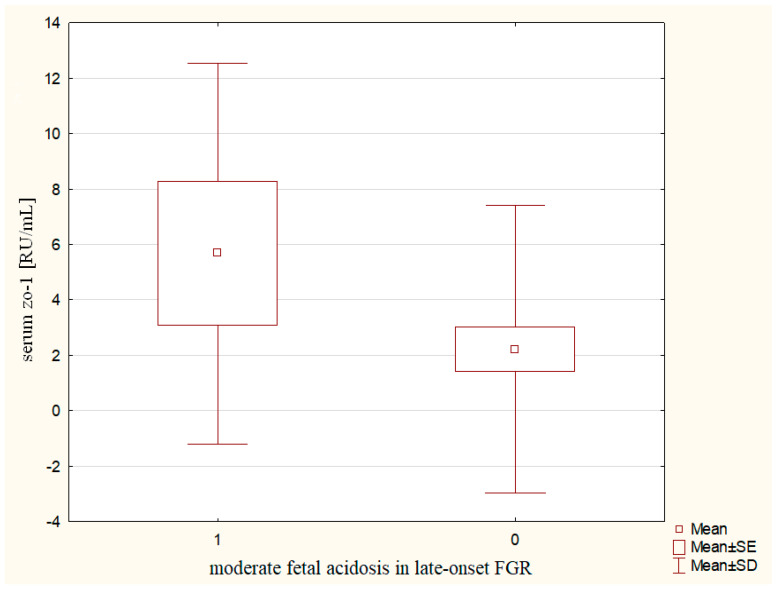
The maternal serum zo-1 concentrations in relation to moderate fetal acidosis in the late-onset FGR.

A significant relationship between serum zo-1 concentrations and moderate fetal acidosis was found in pregnancies complicated by late-onset FGR. The mothers whose newborns developed moderate acidosis had significantly higher serum zo-1 levels (5.67 ± 6.87 vs. 2.21 ± 5.18 RU/mL, *p* ~ 0.0471) as compared to the late-onset FGR pregnancies without moderate acidosis after delivery (Figure 5). In addition, the serum zo-1 concentrations may serve as a useful biomarker in the prediction of moderate acidosis in late-onset FGR neonates (cut-off value for zo-1: 0.157 RU/mL, sensitivity 0.86, specificity 0.78, PPV 0.40, NPV 0.96, AUC 0.747, *p* ~ 0.0380).

## 3. Discussion

### 3.1. The Molecular Indicators of Blood–Brain Barrier Disintegration

There are no data in the literature about blood–brain barrier integrity and TJ organization in human pregnancy complicated by FGR. Our previous observations showed significantly higher serum levels of CLN5 and CLN5/zo-1 ratio, which coexisted with the higher release of S100B into maternal circulation [33]. The serum CLN5/zo-1 index was also statistically higher in the FGR group with a brain-sparing effect than FGR without circulatory centralization, which also co-occurred with the higher serum NME1 levels [34]. Moreover, higher serum NME1 concentrations were observed in relation to fetal distress, newborn neurological disorders, and IVH in the FGR group [33]. The lack of significant differences in serum TJ proteins concentrations between the early- and late-onset FGR pregnancy allows us to speculate that even if the blood–brain barrier is disintegrated in FGR, as reported previously [33,34], its breakdown is not dependent on the advancement of the pregnancy.

Despite the comparable serum TJ protein levels in both groups, the mothers of newborns with the early-onset FGR, who developed IVH postnatally, had significantly higher OCLN concentrations during pregnancy. Kaźmierski et al. observed higher serum levels of OCLN, S100B, and the CLN5/zo-1 ratio in ischemic stroke patients with a clinical deterioration caused by hemorrhagic transformation [35], which indicates the similarities among the processes involved in the blood–brain barrier breakdown in adults with adverse neurological events, and the changes of the blood–brain barrier permeability in pregnancies complicated by FGR. Winkler et al. also reported the role of TJ proteins in the maintenance of blood–brain barrier integrity. The researchers found a downregulated CLN1, CLN3, and OCLN after middle cerebral artery occlusion and reperfusion or hypoxia of isolated brain vessels. In mice with the knockout of CLN3 expression, the CLN1 was reduced. Moreover, the CLN5 and OCLN were decreased in the brain areas with CLN3 knockout. The authors suggested that TJ modulation may improve the prognosis of stroke [36].

### 3.2. The Molecular Indicators of Neuronal Injury

Anantha et al. reported the role of the growth differentiation factor-5 (GDF5), which is a dopaminergic neurotrophic factor, in the upregulation of the NME1 expression in adult rat brains, in vivo. Moreover, the expression of the NME1 and serine threonine receptor-associated protein kinase mRNAs was proven in the developing and adult rodent midbrain. The researchers showed that treatment with NME1 can promote neurite growth in SH-SY5Y cells and in a culture of dopaminergic neurons treated with the neurotoxin 6-hydroxydopamine. A similar effect of NME1 was observed in SH-SY5Y neuronal cell lines and dopaminergic neurons with overexpression of the human wild-type α-synuclein and in stable SH-SY5Y cell lines with the G2019S LRRK2 mutation. Moreover, the enhanced transcription dependent on the nuclear factor kappa-light-chain-enhancer of activated B cells (NF-κB) was involved in the neurite growth-promoting effect of NME1. The researchers concluded that NME1 improves mitochondrial function, which is disturbed in Parkinson’s disease, and should be considered as a potential therapeutic factor for axonal protection [37,38]. The NME1 was also reported as a suppressor of the mutant huntingtin toxicity and its aggregation [39]. Considering that GDF5 may protect against α-synuclein-induced dopaminergic neuron degeneration in vivo [40] and that GDF5 causes the increased expression of NME1 in dopaminergic neurons in vivo [38], NME1 could protect against degeneration induced by α-synuclein [41].

The serum NME1 concentrations are higher in the FGR group with brain-sparing effect [34] and are increased in relation to fetal distress and neurological disorders, which may be useful in the prediction of IVH among FGR newborns [33]. As reported previously, the NME1 was suggested to have a neuroprotective role and occurred only in the culture of damaged neurons [42], which is important considering that the levels of this protein do not differ among early-onset and late-onset FGR pregnancies. It allows us to suppose that despite the higher IVH occurrence in the early-onset FGR in our study, the late-onset FGR is also at risk for neurodevelopmental disorders, but it is not determined by the gestational age. The DIGITAT study revealed that birthweight below the 2.3^rd^ percentile is the strongest prognostic factor of impaired neurodevelopment in fetuses born between 36–41 weeks. Van Wyk et al. concluded that expectant management may negatively influence birthweight and neurodevelopment in 2-year-old children [43].

The significantly higher serum NME1 concentrations in the early-onset FGR group were associated with fetal circulatory centralization and preterm delivery. Such correlations were not found in the late-onset FGR group, which may confirm the previously reported neuroprotective role of NME1 [44] in pregnancies complicated by early-onset FGR. In addition, the usefulness of serum NME1 concentrations in the prognosis of extremely low birth weight was set with a sensitivity of 66% and a specificity of 88% in the early-onset FGR. The cut-off value for NME1 concentration for this adverse perinatal outcome was 38.97 pg/mL, which is higher than the threshold for prediction of the circulatory centralization in our study (26.31 pg/mL) but still lower than the predictive cut-off for prognosis of IVH (40.45 pg/mL) [33]. The increase in the cut-off value for NME1, along with the severity of predicted perinatal outcomes, may confirm its utility as a biomarker in the monitoring and management of FGR pregnancies.

Despite the fact that S100B concentrations did not differ between study groups, significantly higher S100B concentrations were observed in late-onset FGR pregnancies in relation to fetal distress. Although the S100B concentrations are higher in FGR as compared to physiological gestation [33] and are not dependent on the brain-sparing effect among growth-restricted fetuses [34], this neuronal injury marker does not differentiate the early- and late-onset FGR pregnancy.

Despite the previously reported role of the *N*-methyl-d-aspartate receptors (NMDA) in the early stages of development [45], there was no difference in its NR1 subunit between early- and late-onset FGR. In addition, our previous studies did not confirm the usefulness of NR1 evaluation as a molecular indicator of brain injury among growth-restricted fetuses [33,34]. Further research is needed, engaging the other subunits of this receptor and the relationship of its subunits, whose reduction was reported in growth-restricted rats due to hypoxia-ischemia placental insufficiency [46,47].

### 3.3. Adverse Neurological Outcomes in FGR Newborns

Alves de Alencar Rocha et al. examined whether early- and late-onset FGR alters the brain development differentially. For this purpose, they performed an occlusion of a single umbilical artery and induced placental insufficiency in pregnant sheep. The researchers found that the early-onset FGR fetuses became progressively hypoxic over the first 10 days after the onset of placental failure, whereas the late-onset FGR fetal sheeps were significantly hypoxic as compared to healthy controls from the first day after the beginning of the placental insufficiency. Moreover, the authors observed the white matter injury in the early-onset FGR fetuses, and a reduction in 2′-3′-cyclic nucleotide 3′-phosphodiesterase (CNPase) positive and myelin basic protein (MBP) positive density in the periventricular white matter, subcortical white matter, intragyral white matter, subventricular zone, and external capsule in comparison to control group. Furthermore, they noticed a decreased number of mature oligodendrocytes and proved the neuroinflammatory processes in early-onset FGR sheep with reactive astrogliosis in the intragyral white matter and cortex, with coexisting increased amounts of ionized calcium binding adaptor molecule 1 (Iba1) positive activated microglia in the periventricular white matter, subventricular zone, and cortex. The late-onset FGR was related to a decrease in CNPase positive myelin expression and a reduced count of mature oligodendrocytes in the white matter. Furthermore, the late-onset FGR induced caspase-3-positive apoptosis within the cortex. These data points, representing the pregnancy advancement at the time of the growth retardation onset, is an important factor of altered brain development in FGR sheep [48]. Fetal neurodevelopment concerns many processes, of which the blood–brain barrier integrity and the TJ are considered. Despite the data on animal models, we observed no changes in the blood–brain barrier tightness among the early- and late-onset FGR fetuses.

Yawno et al. observed the cerebellum neuropathology in FGR lambs after birth with a ~18% reduction in the count of granule cell bodies within the internal granular layer and a ~80% decrease in neuronal branching and extension within the molecular layer. The 8-hydroxy-2′-deoxyguanosine immunoreactivity as an oxidative stress marker was significantly higher in FGR lambs within the molecular layer and the white matter in comparison to healthy controls. The structural integrity of neurons was already aberrant in the FGR cerebellum at 115 and 124 days of gestation, respectively. The inflammatory cells were significantly upregulated and the permeability of the blood–brain barrier was increased [49]. Similarly, Castillo-Melendez et al. suggested that FGR in lambs leads to cerebrovascular changes in the white matter and causes cerebrovascular deficits, such as reduced area of the basal lamina, decreased levels of vascular endothelial growth factor, lower endothelial cells content, and higher blood–brain barrier permeability [50,51], which is compatible with our previous observations [33,34].

The identified risk factors for IVH include small birth weight, lack of antenatal corticosteroids, maternal chorioamnionitis, 5-min Apgar score < 5 points, umbilical cord pH < 7, respiratory distress syndrome, early onset sepsis, hypercapnia, pCO_2_ fluctuations, prolonged intubation, inhaled nitric oxide, inotropes or normal saline boluses, metabolic derangements, opioids infusions and bicarbonate/THAM therapy, persistent ductus arteriosus treatment, thrombocytopenia, cardiopulmonary resuscitation, intubation at delivery, and others [52,53,54]. Both the early-onset and late-onset FGR are significant risk factors for cerebral palsy [22,23,24] and FGR is also an independent risk factor for IVH. A number of researchers have independently demonstrated an association between placental insufficiency with abnormal umbilical artery blood flow and the occurrence of IVH, periventricular leukomalacia, and lesions in the basal ganglia [17,55,56]. Bernstein et al. observed a nonsignificant trend toward an increase in the incidence of IVH in FGR neonates, born between 25–30 weeks of gestation. When the researchers divided the study group according to delivery weeks, they noticed a significantly lower frequency of IVH in FGR neonates born before 28 weeks as compared to those born prematurely without growth disorders. At the same time, the incidence of IVH increased significantly in FGR neonates, delivered after the 34th weeks of pregnancy [22,57,58,59], which may confirm the greater sensitivity of mature fetuses to intrauterine hypoxia. Baschat, Eixarch et al. and Oros et al. independently observed that children with a prenatal diagnosis of late-onset FGR and circulatory centralization, born prematurely or at term, presented neurobehavioral abnormalities in the neonatal period and at the age of two years. The neurodevelopment of newborns diagnosed with early-onset FGR was abnormal in motor, cognitive and behavioral functions, but these findings may have been interfered by implications of prematurity [22,60,61,62]. Lubrano et al. observed IVH in 0.8% and 1.7% of early-onset and late-onset FGR, respectively [63], whereas Mileusnić-Milenović noticed a higher incidence of IVH—44.3% of FGR neonates developed 1st stage of IVH, while 4.3% of them had 2nd stage of IVH. None of the newborns developed the 3rd or 4th IVH stage [64]. Contrary to the cited research, the majority of IVH diagnoses in our study involved early-onset FGR newborns (15.4%), and only 2.0% referred to late-onset FGR. This may be due to proper monitoring and management of the FGR pregnancy and the decision-making about the term of delivery to avoid neurodevelopmental disorders. Despite the low occurrence of IVH in the late-onset FGR group, it is also at risk of developing neurodevelopmental disorders. The researchers reported the changed brain metabolites in the late-onset small for gestational age (SGA) fetuses [65] and in the late FGR newborns [66]. Moreover, the meta-analysis of SGA children revealed up to 0.32 standard deviation poorer neurodevelopmental scores [67]. Furthermore, Baschat et al. revealed that no Doppler parameter may serve as an independent contributor to IVH [17], which is why it is necessary to search for maternal serum markers that would enable monitoring of pregnancies complicated by FGR and to determine the appropriate term of delivery, so as to achieve a compromise between avoiding neurological disorders and, at the same time, complications of prematurity.

Within the neurological complications among FGR newborns, the periventricular leukomalacia was also noticed, but its incidence was low in our study and did not differ between the studied groups.

### 3.4. Placental TJ Proteins Expression

To the best of our knowledge, TJ protein expression has not been evaluated in human FGR pregnancy until now. Although higher placental CLN5 expression has been observed in FGR pregnancy as compared to physiological gestation as we reported previously [33], TJ protein expression did not differ between the early- and late-onset FGR, which could prove that changes in placental cell tightness do not depend on the advancement of pregnancy at the time of FGR diagnosis. Moreover, the TJ proteins expression was also comparable between FGR with and without brain-sparing effect [34]. The neurological complications in FGR, including IVH, were associated with a decreased CLN4 expression, which is useful in the prognosis of IVH [33]. Moreover, the IVH in FGR newborns is related to the inhibition of CLN4 expression, which may have a predictive value for the IVH prognosis in FGR pregnancy with a brain-sparing effect with a sensitivity of 100% and specificity of 83% [34]. Similar observations were not found in early-onset and late-onset FGR, which may indicate a greater role of circulatory centralization in the development of these complications than the advancement of pregnancy at the time of the FGR diagnosis. Furthermore, Ahn et al. reported that CLN4 was predominantly expressed in mouse placental tissue and is responsible for a decrease in paracellular communication and conduction through a selective reduction in Na^+^ ions permeability [68], which underlines the role of CLN4 in placental TJ organization.

Marzioni et al. reported that zo-1 and OCLN are key TJ components. They noticed that fetal vessels are positive in immunostaining for zo-1 throughout pregnancy, while for OCLN is mainly at term. Moreover, the researchers observed the changeable pattern of immunoreactivity in regard to localization, and showed that cytotrophoblastic cells, located distally to the villous stroma, were negative for these TJ proteins. Furthermore, the changeable pattern of immunostaining in hydatidiform moles suggests its participation in normal placenta development as well as maintaining the TJ structure and functions. On the contrary, our study revealed the OCLN expression within all evaluated gestational periods, and its similar expression regardless of the FGR advancement [69]. Leach et al. observed that the term human placentas from singleton pregnancies demonstrates a differential surface TJ proteins expression. Within the same placental villous tree, arteries, veins, and arterioles were immunopositive for OCLN, but the exchange vessels in terminal villi showed negative immunostaining, whereas the zo-1 was observed everywhere. Moreover, no significant differences in frequency, position, or dimension of TJ were found [70].

The only study on animal FGR model in gilts was performed by Guidoni et al., who noticed a lower CLN1 intensity mainly in the fetal epithelium and maternal endothelial cells in FGR, meconium-stained, and non-FGR fetuses infected with porcine reproductive and respiratory syndrome virus 2 (PRRSV2) as compared to healthy controls. Furthermore, the CLN4 immunoreactivity was decreased in maternal endothelial cells of the FGR group as compared to the control and meconium-stained group. Moreover, the zo-1 intensity was significantly lower in maternal and fetal epithelia of placentae within FGR, non-FGR, meconium-stained fetuses in comparison to non-infected control group. The lower CLN1, CLN4, and zo-1 intensity in PRRSV2 infected groups as compared to the control group provide potential evidence of altered TJ permeability, allowing the virus to cross through the maternal-fetal barrier and show that the TJ organization differs between animal FGR group and healthy controls [71].

Zhang et al. observed a decreased zo-1 and CLN4 expression in preeclamptic placentae at the mRNA and protein levels as compared to physiological pregnancy. On the other hand, the CLN8 expression was increased in pregnancy complicated by preeclampsia in comparison to healthy controls, whereas no differences in OCLN expression were observed. The altered expression of TJP in preeclamptic patients was similar to that observed in hypoxic conditions, which may suggest that hypoxia-induced TJ dysfunction may be engaged in the pathogenesis of preeclampsia. Furthermore, the researchers evaluated the HIF1α expression in preeclamptic and healthy placentae, and noticed a significant increase of HIF1α expression in preeclamptic patients, so they concluded that hypoxia may underlie the etiology of preeclampsia through cellular signaling pathways in which HIF1α participates [72]. Although Lievano et al. observed no differences in OCLN and zo-1 placental immunostaining in preeclampsia, the expression of CLN1, CLN3, and CLN5 decreased, which may suggest the TJ unsealing, possibly as a result of decreased perfusion [73]. Because of the similar etiology between preeclampsia and FGR, related to hypoxia and placental insufficiency, searching for the changed placental TJ protein expression seems to be justified.

## 4. Methods and Materials

The study was performed in the Department of Perinatology and Gynecology, Gynecological and Obstetric Hospital of the Medical University in Poznan. The serum assays and placental expression were evaluated in the Department of Neurochemistry and Neuropathology of the Medical University in Poznan. The project obtained the approval of the Bioethics Committee (issue 667/15, 11 June 2015, annex issue 787/17, 22 June 2017). The research was conducted with appropriate ethics and scientific principles in compliance with the Helsinki Declaration. All patients signed an informed consent for the serum and placental assays.

### 4.1. The Studied Groups

The early-onset FGR group included 39 women, while the late-onset FGR group was composed of 51 females. The diagnosis of FGR was based on the Figueras and Gratacós criteria [1]. Early-onset FGR was recognized until 32 weeks of gestation, and late-onset FGR was above 32 weeks of gestation [74]. A medical interview concerns the obstetric history, the course of pregnancy, medications, and chronic diseases, including any central nervous system pathology or disorders that may affect the blood–brain barrier integrity. No clinical symptoms of central nervous system diseases were noticed in pregnant women included in the study. All females had a gynecological examination and a fetal ultrasound with Doppler imaging (GE Voluson E10 BT18). The ultrasound measurements included the estimated fetal weight, the estimated fetal weight percentile, the amniotic fluid index, the umbilical artery pulsatility index, the presence of an umbilical artery absent end-diastolic flow or an umbilical artery reversed end-diastolic flow, the middle cerebral artery pulsatility index, the uterine arteries pulsatility indexes, the ductus venosus pulsatility index, and the presence of umbilical venous pulsations. The estimated fetal weight and its percentile were calculated based on the Hadlock formula [75,76]. The amniotic fluid abnormalities were identified in regard to the Magann et al. percentile charts [77]. The cerebroplacental ratio quantified a redistribution of cardiac output by dividing Doppler indices—middle cerebral artery pulsatility index and umbilical artery pulsatility index, and the result was referred to the Baschat and Gembruch percentile charts [78]. In the uterine artery score, one point was assigned to each abnormal parameter—high pulsatility index and presence of notch in reference to Sekizuka and Gudmundsson [79,80]. The birth weight was divided into low (1500–2500 g), very low (1000–1500 g), and extremely low (<1000 g) [81]. Metabolic acidosis was described as mild (pH 7.20–7.29), moderate (pH 7.10–7.19), severe (pH 7.00–7.09), and extremely severe (pH < 7.00) [82]. When the 1-min Apgar scored below 10 points, the 3-min result was also evaluated. Women were excluded from the study in any case of confirmed malnutrition, smoking, alcohol consumption, drug intake, treatment with antiepileptic drugs, warfarin, anticancer drugs, folic acid antagonists, cyanotic congenital heart defects, heart failure, uncontrolled asthma, chronic obstructive pulmonary disease, cystic fibrosis, diabetes mellitus, renal failure, nephrotic syndrome, renal transplantation, systemic lupus erythematosus, antiphospholipid syndrome, Crohn’s disease, ulcerative colitis, severe anemia, sickle cell anemia, beta-thalassemia, hemoglobin H disease, and uterine malformations. The pregnancies suspected and confirmed postdelivery as chromosomal and autosomal abnormalities, uniparental disomies, microdeletions, congenital anomalies, and congenital infections were excluded from the study. The placental factors that disqualified the patients included placenta previa, placenta accreta, placental infarcts, placental villous thrombosis, and placental tumors.

### 4.2. The Blood and Placental Samples Collection

In total, 7.5 mL of whole blood was withdrawn from the mother’s peripheral vein and collected into Monovette tubes without anticoagulant. The samples were centrifuged for 10 min at 2700× *g*, and then the serum was placed into Eppendorfs and frozen at −80 °C. The placental sample, with the size of 25 cm^2^, was taken postdelivery and frozen at −80 °C.

### 4.3. The Serum Assays

The commercial ELISA kits were used to evaluate the serum levels of NR1 (Human Glutamate [NMDA] receptor subunit zeta-1, GRIN1 ELISA Kit, MyBioSource, San Diego, CA, USA), NME1 (Human Nucleoside diphosphate kinase A, NME1 ELISA Kit, MyBioSource, San Diego, CA, USA) and S100B (S100B human ELISA Kit, DRG MedTek, Warsaw, Poland). Because of the unavailability of commercial tests to measure the serum OCLN, zo-1, and CLN5 concentrations, these proteins were assessed by an in-house ELISA method, developed in the Department of Neurochemistry and Neuropathology, Medical University in Poznan. The rabbit anti-human (Occludin Polyclonal Antibody, Zymed, San Francisco, CA, USA, AB_2533977, ZO-1 Polyclonal Antibody, Zymed, San Francisco, CA, USA, AB_2533938) and mouse anti-human antibodies (Occludin Monoclonal Antibody (OC-3F10), Invitrogen, Waltham, MA, USA, AB_2533101, ZO-1 Monoclonal Antibody (ZO1-1A12), Invitrogen, Waltham, MA, USA, AB_2533147) were used for the capturing and detection of OCLN and zo-1. The CLN5 levels were measured using the mouse anti-human antibodies (Claudin 5 Monoclonal Antibody (4C3C2), Zymed, San Francisco, CA, USA, AB_2533200) for the capture and rabbit anti-human antibodies (Claudin 5 Polyclonal Antibody, Abcam, Cambridge, UK, AB_2533157) in detection. The goat anti-mouse IgG (Goat anti-Mouse IgG (H+L) Cross-Adsorbed Secondary Antibody, HRP, Invitrogen, Waltham, MA, USA, AB_2536527) served as secondary antibodies for OCLN and zo-1. Similarly, in serum CLN5 assays, the goat anti-rabbit antibodies (H+L, HRP, Invitrogen, Waltham, MA, USA) were used. For all measurements, the Substrate Reagent Pack (Substrate Reagent Pack, R&D Systems, Minneapolis, MN, USA) was applied. As standards served the recombinant proteins, respectively, for OCLN (Recombinant Human Occludin GST (N-Term) Protein, Novus Biologicals, Littleton, CO, USA) and CLN5 (Recombinant Human Claudin-5 GST (N-Term) Protein, Novus Biologicals, Littleton, CO, USA). Due to the unavailability of the standard for zo-1, the relative units (RU) were determined, based on optical density (OD) at 450 nm, as the quotient: 10 samples OD/cut-off OD. The OD value was calculated from the serum zo-1 levels of 48 healthy patients, and the 95th percentile was the cut-off. The serum zo-1 concentration was given in RU/mL, and the levels of the other biochemical measurements were expressed in pg/mL. The Nunc MaxiSorp^TM^ plates (ThermoFisher, Waltham, MA, USA), an automated RT-3100 microplate washer (Rayto Life and Analytical Sciences Co., Ltd., Shenzhen, China) and an ELx800 microplate reader (BioTek, Winooski, VT, USA) were used in all ELISA procedures. The CLN5/zo-1 index was counted to assess the changes of the blood–brain barrier integrity.

### 4.4. The Placental TJP Expression

The placental expression of CLN5, OCLN, and zo-1 was assessed by in-house ELISA, developed in the Department of Neurochemistry and Neuropathology, Medical University in Poznan. Moreover, the commercial ELISA assay was used to evaluate the placental CLN4 expression (ELISA Kit for Claudin 4 (CLDN4), USCN Life Science, Wuhan, China). Firstly, the placental samples were homogenized in a 1-litre buffer solution containing 150 millimoles (mM) NaCl (Sigma Aldrich, Saint Louis, MO, USA), 5 mM ethylenediaminetetraacetic acid (Sigma Aldrich, Saint Louis, MO, USA), and 50 mM Tris buffer solution (Sigma Aldrich, Saint Louis, MO, USA). Then the mixture of protease inhibitors (Protease Inhibitor Tablets For General Use, Sigma Aldrich, Saint Louis, MO, USA) and Triton X-100 (Sigma Aldrich, Saint Louis, MO, USA) was added until the solution’s concentration reached 1%. The enzyme inhibitors contained fluorinated 4-(2-aminoethyl) benzenesulfonyl hydrochloride, aprotinin, bestatin hydrochloride, trans-epoxysuccinyl-l-leucylamido (4-guanidino) butane, ethylenediaminetetraacetic acid, and leupeptin hemisulfate. Subsequently the homogenate was centrifuged for 15 min at 2750× *g*, and the filtrate served for TJ proteins expression assays. All ELISA procedures were performed using an automated microplate washer (RT-3100 Microplate Washer, Rayto Life and Analytical Sciences Co., Ltd., Shenzhen, China), and the results were read out in an ELx800 microplate reader (Absorbance Microplate Reader, BioTek, Winooski, VT, USA). The Lowry method was used for TJ protein quantification [83], and the placental expression was given in ng/mg total protein.

### 4.5. The Newborns Neurological Examination

All newborns underwent a neurological examination. The evaluation concerned the level of alertness, the cranial nerves function, the sensory and motor examination, the presence of reflexes, also the primitive ones. The infants born prematurely before 35 weeks of gestation had the first transtemporal ultrasound until the third day after delivery and the second between the fifth and the seventh postpartum day. The diagnosis of IVH was set with reference to the Papile classification [84]. Further management depended on the changes found in the first cranial sonography. In all cases of questionable or incorrect ultrasound imaging, magnetic resonance imaging (MRI) was applied. In the absence of abnormalities in transtemporal ultrasound, the MRI was performed between 38 and 42 postconceptional weeks. The infants born after 35 gestational weeks, recognized with an umbilical artery pH below 7.0 or an Apgar score less than or equal to 3 points, with subsequent therapeutic hypothermia, had a transtemporal Doppler ultrasound, and the MRI between the seventh and the tenth-day postdelivery. If the targeted hypothermia management was not necessary, the transcranial ultrasound was carried out up to the third day postpartum and then between the fifth and the seventh day. In cases suspected of neurological abnormalities, the MRI was commissioned, which allowed to confirm or exclude periventricular leukomalacia [85,86].

### 4.6. Statistical Analysis

The statistical analysis was performed with Statistica StatSoft 13.1 (StatSoft, Kraków, Poland) and MedCalc 20.113 (MedCalc Statistical Software Ltd., Milanówek, Poland). If the normality of distribution was confirmed by the use of the Kolmogorov–Smirnov, Lilliefors, and Shapiro–Wilk evaluations, the Student’s *t*-test was performed, otherwise the non-parametric Mann–Whitney U test was chosen. The Chi-squared and Fisher’s exact tests were used for analyses on a nominal scale. The receiver operating characteristic curves were determined using DeLong’s non-parametric method and described as the cut-off value, sensitivity, specificity, positive predictive value (PPV), negative predictive value (NPV), and the area under the curve (AUC). The significance level was assumed as 0.05.

## 5. Conclusions

Blood–brain barrier disintegration is not dependent on the pregnancy advancement at the time of FGR diagnosis. In the spectrum of neonatal complications, IVH has been observed in association with higher OCLN release into maternal blood.

Perinatal observation of biomarkers in FGR showed that the early-onset FGR is associated with a higher release of NME1 into the maternal circulation in relation to brain-sparing effects, and premature delivery. In addition, in the late-onset FGR, higher release of S100B into maternal serum is observed, associated with fetal distress. Furthermore, there is a higher release of zo-1 into maternal circulation with regard to newborns’ moderate acidosis in the late-onset FGR group.

NME1 may serve as a biomarker useful in the prediction of fetal circulatory centralization and extremely low birth weight in pregnancies complicated by the early-onset FGR. Moreover, the serum zo-1 concentration may have a prognostic value of moderate neonatal acidosis in late-onset FGR pregnancies.

## Figures and Tables

**Table 1:** The characteristics of the early-onset and late-onset FGR group.

Parameters	Early-Onset FGR (*n* = 39)	Late-Onset FGR (*n* = 51)	*p*-Value
Age (years; mean ± SD)	29 ± 3	29 ± 5	0.9666
BMI at the first prenatal visit (kg/m^2^, median, min–max)	22.3 (15.8–42.0)	22.2 (15.2–38.1)	0.7792
Gestational weight gain (kg; median, min–max)	12.0 (-0.5–25.0)	13.0 (3.0–32.0)	0.6953
The pregnancy advancement at the time of FGR diagnosis (weeks; median, min–max)	30 (22–31)	36 (32–40)	<0.0001
Gravidity (median, min–max)	1 (1–5)	1 (1–7)	0.9453
Parity (median, min–max)	0 (0–3)	0 (0–4)	0.5868

**Table 2:** Ultrasound findings in pregnancy complicated by the early-onset and late-onset FGR.

Parameters	Early-Onset FGR (*n* = 39)	Late-Onset FGR (*n* = 51)	*p*-Value
Estimated fetal weight (g; median, min–max)	1265 (439–2166)	2211 (1290–2920)	0.0001
Percentile of estimated fetal weight (median, min–max)	0.1 (0.1–9.0)	2.0 (0.1–9.0)	0.0001
Amniotic fluid index (cm, mean ± SD)	8.9 ± 3.7	8.7 ± 3.8	0.8003
Oligohydramnion (%)	51.3	39.2	0.2536
Polyhydramnion (%)	2.6	0.0	0.4333
Umbilical artery pulsatility idex (UA PI) (median, min–max)	1.3 (0.8–3.0)	1.0 (0.6–2.2)	0.0001
Abnormal UA PI (%)	59.0	27.5	0.0052
Umbilical artery absent end-diastolic flow (%)	23.1	3.9	0.0084
Umbilical artery reversed end-diastolic flow (%)	5.1	0.0	0.1850
Middle cerebral artery pulsatility index (MCA PI) (median, min–max)	1.3 (0.8–2.5)	1.3 (0.8–2.3)	0.6971
Abnormal MCA PI (%)	48.7	39.2	0.4922
Cerebroplacental ratio (median, min–max)	1.0 (0.4–2.6)	1.4 (0.4–3.2)	0.0075
The onset of fetal circulatory centralization (weeks; median, min–max)	30.0 (24.0–36.0)	36.5 (33.0–39.0)	<0.0001
Frequency of fetal circulatory centralization (%)	64.1	31.4	0.0028
Left uterine artery pulsatility index (LUtA PI) (median, min–max)	1.2 (0.5–3.2)	0.8 (0.5–1.9)	0.0084
Abnormal LUtA PI (%)	56.4	21.6	0.0015
Right uterine artery pulsatility index (RUtA PI) (median, min–max)	1.1 (0.4–3.9)	0.9 (0.3–3.7)	0.0725
Abnormal RUtA PI (%)	53.9	17.7	0.0007
Uterine artery score (points; median, min–max)	1 (0–4)	0 (0–4)	0.0027
Ductus venosus pulsatility index (DV PI) (median, min–max)	0.7 (0.2–1.4)	0.5 (0.2–1.6)	0.0866
Abnormal DV PI (%)	25.6	7.8	0.0373
Umbilical vein pulsations (%)	2.6	0.0	0.4333

**Table 3:** Serum measurements in pregnancies complicated by the early-onset and late-onset FGR.

Serum Measurements	Early-Onset FGR (*n* = 39)	Late-Onset FGR (*n* = 51)	*p*-Value
NR1 (pg/mL; mean ± SD)	1121.13 ± 1959.42	1424.96 ± 3382.32	0.5896
NME1 (pg/mL; mean ± SD)	208.66 ± 990.55	40.70 ± 83.36	0.8543
S100B (pg/mL; mean ± SD)	33.36 ± 42.59	26.77 ± 39.35	0.4692
OCLN (pg/mL; mean ± SD)	12.47 ± 62.09	47.94 ± 142.89	0.2457
CLN5 (pg/mL; mean ± SD)	63.63 ± 151.02	82.10 ± 179.95	0.6238
zo-1 (RU/mL; mean ± SD)	2.13 ± 2.90	2.95 ± 5.55	0.5715
CLN5/zo-1 (mean ± SD)	229.75 ± 899.98	15.80 ± 19.90	0.5905

**Table 4:** The placental TJ proteins expression in pregnancy, complicated by the early-onset and late-onset FGR.

Placental Expression	Early-Onset FGR (*n* = 39)	Late-Onset FGR (*n* = 51)	*p*-Value
OCLN (ng/mg total protein; mean ± SD)	0.21 ± 0.16	0.17 ± 0.16	0.2628
CLN5 (ng/mg total protein; mean ± SD)	0.02 ± 0.04	0.01 ± 0.01	0.7712
CLN4 (ng/mg total protein; mean ± SD)	0.14 ± 0.12	0.17 ± 0.09	0.3647
zo-1 (ng/mg total protein; mean ± SD)	0.27 ± 0.20	0.23 ± 0.15	0.7054

**Table 5:** The perinatal outcomes among women in pregnancy, complicated by the early-onset and late-onset FGR.

Parameters	Early-Onset FGR (*n* = 39)	Late-Onset FGR (*n* = 51)	*p*-Value
Intrauterine fetal death (%)	5.1	0.0	0.1850
The term of delivery (weeks; median, min–max)	33.5 (26.0–39.0)	38.0 (33.0–41.0)	<0.0001
Premature delivery (%)	69.2	10.0	<0.0001
Mode of delivery (%)			
Spontaneous	18.4	25.5	0.4561
Cesarean section	81.6	66.7	0.1498
Vacuum extraction	0.0	7.8	0.1326
Forceps	0.0	0.0	-
Fetal distress (%)	64.1	39.2	0.0184
Blood loss (mL; median, min–max)	400 (100–1200)	400 (100–1200)	0.8669
Placental weight (g; median, min–max)	371 (162–698)	440 (245–580)	0.1403

**Table 6:** Perinatal outcomes in the early-onset and late-onset FGR newborns.

Parameters	Early-Onset FGR (*n* = 39)	Late-Onset FGR (*n* = 51)	*p*-Value
Birth weight (g; median, min–max)	1400 (420–2860)	2485 (1260–3080)	<0.0001
1500–2500 (%)	41.0	49.0	0.5242
1000–1500 (%)	15.4	2.0	0.0398
<1000 (%)	35.9	0.0	<0.0001
Apgar (points; median, min–max)			
1st minute	8 (0–10)	10 (4–10)	<0.0001
3rd minute	8 (2–10)	8 (6–9)	0.5233
5th minute	9 (4–10)	10 (8–10)	<0.0001
pH (median, min–max)			
venous	7.32 (7.19–7.42)	7.33 (7.01–7.46)	0.3808
arterial	7.27 (6.99–7.40)	7.29 (6.95–7.45)	0.8505
BE (mEq/L; median, min–max)			
venous	−2.6 (−10.4–1.1)	−2.5 (−11.3–3.2)	0.3889
arterial	−2.0 (−11.9–1.0)	−2.4 (−13.4–3.4)	0.6855
Metabolic acidosis (%)			
pH < 7.30 overall	59.0	54.9	0.8304
7.20–7.29	46.2	39.2	0.5266
7.10–7.20	10.3	13.7	0.7508
7.00–7.09	0.0	0.0	-
<7.00	2.6	2.0	1.0000
Intraventricular hemorrhage (%)	15.4	2.0	0.0398
1st grade	10.3	2.0	0.1620
2nd grade	2.6	0.0	0.4333
3rd grade	2.6	0.0	0.4333
4th grade	0.0	0.0	-
Periventricular leucomalacia (%)	5.1	0.0	0.1850
Respiratory distress syndrome (%)	33.3	2.0	<0.0001
1st grade	18.0	2.0	0.0191
2nd grade	7.7	0.0	0.0778
3rdgrade	7.7	0.0	0.0778
4th grade	2.6	0.0	0.4333
Respiratory failure (%)	38.5	3.9	0.0001
Congenital pneumonia (%)	5.1	0.0	0.1850
Bronchopulmonary dysplasia (%)	5.1	0.0	0.1850
Necrotizing enterocolitis (%)	5.1	0.0	0.1850
Retinopathy (%)	5.1	2.0	0.5767
Death during hospitalization (%)	2.6	0.0	0.4333

## Data Availability

The data presented in the study are available from the corresponding author upon reasonable request.

## References

[1] Figueras F., Gratacós E. (2014). Update on the diagnosis and classification of fetal growth restriction and proposal of a stage-based management protocol. Fetal Diagn. Ther..

[2] Ferrazzi E., Bozzo M., Rigano S., Bellotti M., Morabito A., Pardi G., Battaglia F.C., Galan H.L. (2002). Temporal sequence of abnormal Doppler changes in the peripheral and central circulatory systems of the severely growth-restricted fetus. Ultrasound Obstet. Gynecol..

[3] Baschat A.A. (2010). Fetal growth restriction—From observation to intervention. J. Perinat. Med..

[4] Savchev S., Figueras F., Sanz-Cortes M., Cruz-Lemini M., Triunfo S., Botet F., Gratacos E. (2014). Evaluation of an optimal gestational age cut-off for the definition of early- and late-onset fetal growth restriction. Fetal Diagn. Ther..

[5] Damodaram M., Story L., Kulinskaya E., Rutherford M., Kumar S. (2011). Early adverse perinatal complications in preterm growth-restricted fetuses. Aust. N. Z. J. Obstet. Gynaecol..

[6] Hecher K., Bilardo C.M., Stigter R.H., Ville Y., Hackelöer B.J., Kok H.J., Senat M.V., Visser G.H.A. (2001). Monitoring of fetuses with intrauterine growth restriction: A longitudinal study. Ultrasound Obstet. Gynecol..

[7] Schneider H. (2009). Tolerance of human placental tissue to severe hypoxia and its relevance for dual ex vivo perfusion. Placenta.

[8] Dall’Asta A., Brunelli V., Prefumo F., Frusca T., Lees C.C. (2017). Early onset fetal growth restriction. Matern. Health Neonatol. Perinatol..

[9] Figueras F., Caradeux J., Crispi F., Eixarch E., Peguero A., Gratacos E. (2018). Diagnosis and surveillance of late-onset fetal growth restriction. Am. J. Obstet. Gynecol..

[10] Crovetto F., Triunfo S., Crispi F., Rodriguez-Sureda V., Dominguez C., Figueras F., Gratacos E. (2017). Differential performance of first-trimester screening in predicting small-for-gestational-age neonate or fetal growth restriction. Ultrasound Obstet. Gynecol..

[11] Mifsud W., Sebire N.J. (2014). Placental Pathology in Early-Onset and Late-Onset Fetal Growth Restriction. Fetal Diagn. Ther..

[12] Oros D., Figueras F., Cruz-Martinez R., Meler E., Munmany M., Gratacos E. (2011). Longitudinal changes in uterine, umbilical and fetal cerebral Doppler indices in late-onset small-for-gestational age fetuses. Ultrasound Obstet. Gynecol..

[13] Giussani D.A. (2016). The fetal brain sparing response to hypoxia: Physiological mechanisms. J. Physiol..

[14] Kady S.M., Gardosi J. (2004). Perinatal mortality and fetal growth restriction. Best Pract. Res. Clin. Obstet. Gynaecol..

[15] Figueras F., Eixarch E., Meler E., Iraola A., Figueras J., Puerto B., Gratacos E. (2008). Small-for-gestational-age fetuses with normal umbilical artery Doppler have suboptimal perinatal and neurodevelopmental outcome. Eur. J. Obstet. Gynecol. Reprod. Biol..

[16] Gardosi J., Madurasinghe V., Williams M., Malik A., Francis A. (2013). Maternal and fetal risk factors for stillbirth: Population based study. BMJ.

[17] Baschat A.A., Gembruch U., Viscardi R.M., Gortner L., Harman C.R. (2002). Antenatal prediction of intraventricular hemorrhage in fetal growth restriction: What is the role of Doppler?. Ultrasound Obstet. Gynecol..

[18] Gabryel B., Kost A., Kasprowska D. (2012). Neuronal autophagy in cerebral ischemia–A potential target for neuroprotective strategies?. Pharmacol Rep..

[19] Malhotra A., Ditchfield M., Fahey M.C., Castillo-Melendez M., Allison B., Polglase G., Wallace E., Hodges R., Jenkin G., Miller S. (2017). Detection and assessment of brain injury in the growth-restricted fetus and neonate. Pediatr. Res..

[20] Rees S., Harding R., Walker D. (2011). The biological basis of injury and neuroprotection in the fetal and neonatal brain. Int. J. Dev. Neurosci..

[21] Baschat A.A. (2011). Neurodevelopment following fetal growth restriction and its relationship with antepartum parameters of placental dysfunction. Ultrasound Obstet. Gynecol..

[22] Miller S.L., Huppi P.S., Mallard C. (2016). The consequences of fetal growth restriction on brain structure and neurodevelopmental outcome. J. Physiol..

[23] Blair E.M., Nelson K.B. (2015). Fetal growth restriction and risk of cerebral palsy in singletons born after at least 35 weeks’ gestation. Am. J. Obstet. Gynecol..

[24] McIntyre S., Taitz D., Keogh J., Goldsmith S., Badawi N., Blair E. (2013). A systematic review of risk factors for cerebral palsy in children born at term in developed countries. Dev. Med. Child. Neurol..

[25] Sweeney M.D., Sagare A.P., Zlokovic B.V. (2018). Blood-brain barrier breakdown in Alzheimer disease and other neurodegenerative disorders. Nat. Rev. Neurol..

[26] Abbott N.J. (2005). Dynamics of CNS barriers: Evolution, differentiation, and modulation. Cell. Mol. Neurobiol..

[27] Ballabh P., Braun A., Nedergaard M. (2004). The blood–brain barrier: An overview: Structure, regulation, and clinical implications. Neurobiol. Dis..

[28] Wolburg H., Lippoldt A. (2002). Tight junctions of the blood–brain barrier: Development, composition and regulation. Vasc Pharmacol..

[29] Kadry H., Noorani B., Cucullo L. (2020). A blood–brain barrier overview on structure, function, impairment, and biomarkers of integrity. Fluids Barriers CNS.

[30] García-Berrocoso T., Penalba A., Boada C., Giralt D., Cuadrado E., Colomé N., Dayon L., Canals F., Sanchez J.-C., Rosell A. (2013). From brain to blood: New biomarkers for ischemic stroke prognosis. J. Proteom..

[31] Saenger A.K., Christenson R.H. (2010). Stroke biomarkers: Progress and challenges for diagnosis, prognosis, differentiation, and treatment. Clin. Chem..

[32] Sharma R., Macy S., Richardson K., Lokhnygina Y., Laskowitz D.T. (2014). A blood-based biomarker panel to detect acute stroke. J. Stroke Cerebrovasc. Dis..

[33] Misan N., Michalak S., Rzymski P., Poniedziałek B., Kapska K., Osztynowicz K., Ropacka-Lesiak M. (2022). Molecular indicators of blood-brain barrier breakdown and neuronal injury in pregnancy complicated by fetal growth restriction. Int. J. Mol. Sci..

[34] Misan N., Michalak S., Kapska K., Osztynowicz K., Ropacka-Lesiak M. (2022). Blood-Brain Barrier Disintegration in Growth-Restricted Fetuses with Brain Sparing Effect. Int. J. Mol. Sci..

[35] Kazmierski R., Michalak S., Wencel-Warot A., Nowinski W.L. (2012). Serum tight-junction proteins predict hemorrhagic transformation in ischemic stroke patients. Neurology.

[36] Winkler L., Blasig R., Breitkreuz-Korff O., Berndt P., Dithmer S., Helms H.C., Puchkov D., Devraj K., Kaya M., Qin Z. (2021). Tight junctions in the blood-brain barrier promote edema formation and infarct size in stroke—Ambivalent effects of sealing proteins. J. Cereb. Blood Flow Metab..

[37] Anantha J., Goulding S.R., Tuboly E., O’Mahony A.G., Moloney G.M., Lomansey G., McCarthy C.M., Collins L.M., Sullivan A.M., O’Keeffe G.W. (2022). NME1 Protects Against Neurotoxin-, α-Synuclein- and LRRK2-Induced Neurite Degeneration in Cell Models of Parkinson’s Disease. Mol. Neurobiol..

[38] Anantha J., Goulding S.R., Wyatt S.L., Concannon R.M., Collins L.M., Sullivan A.M., O’Keeffe G.W. (2020). STRAP and NME1 Mediate the Neurite Growth-Promoting Effects of the Neurotrophic Factor GDF5. iScience..

[39] Wertz M.H., Mitchem M.R., Pineda S.S., Hachigian L.J., Lee H., Lau V., Powers A., Kulicke R., Madan G.K., Colic M. (2020). Genome-wide in vivo CNS screening identifies genes that modify CNS neuronal survival and mHTT toxicity. Neuron.

[40] Goulding S.R., Sullivan A.M., O’Keeffe G.W., Collins L.M. (2020). The potential of bone morphogenetic protein 2 as a neurotrophic factor for Parkinson’s disease. Neural Regen. Res..

[41] Goulding S.R., Anantha J., Collins L.M., Sullivan A.M., O’Keeffe G.W. (2022). Growth differentiation factor 5: A neurotrophic factor with neuroprotective potential in Parkinson’s disease. Neural Regen. Res..

[42] Lööv C., Shevchenko G., Geeyarpuram Nadadhur A., Clausen F., Hillered L., Wetterhall M., Erlandsson A. (2013). Identification of injury specific proteins in a cell culture model of traumatic brain injury. PLoS ONE.

[43] van Wyk L., Boers K.E., van der Post J.A.M., van Pampus M.G., van Wassenaer A.G., van Baar A.L., Spaanderdam M.E.A., Becker J.H., Kwee A., Duvekot J.J. (2012). Effects on (neuro)developmental and behavioral outcome at 2 years of age of induced labor compared with expectant management in intrauterine growth-restricted infants: Long-term outcomes of the DIGITAT trial. Am. J. Obstet. Gynecol..

[44] Romani P., Ignesti M., Gargiulo G., Hsu T., Cavaliere V. (2018). Extracellular NME proteins: A player or a bystander?. Lab Investig..

[45] Waters K.A., Machaalani R. (2004). NMDA receptors in the developing brain and effects of noxious insults. Neurosignals.

[46] Schober M.E., McKnight R.A., Yu X., Callaway C.W., Ke X., Lane R.H. (2009). Intrauterine growth restriction due to uteroplacental insufficiency decreased white matter and altered NMDAR subunit composition in juvenile rat hippocampi. Am. J. Physiol. Regul. Integr. Comp. Physiol..

[47] Phillips T.J., Scott H., Menassa D.A., Bignell A.L., Sood A., Morton J.S., Akagi T., Azuma K., Rogers M.F., Gilmore C.E. (2017). Treating the placenta to prevent adverse effects of gestational hypoxia on fetal brain development. Sci. Rep..

[48] Alves de Alencar Rocha A.K., Allison B.J., Yawno T., Polglase G.R., Sutherland A.E., Malhotra A., Jenkin G., Castillo-Melendez M., Miller S.L. (2017). Early- versus Late-Onset Fetal Growth Restriction Differentially Affects the Development of the Fetal Sheep Brain. Dev. Neurosci..

[49] Yawno T., Sutherland A.E., Pham Y., Castillo-Melendez M., Jenkin G., Miller S.L. (2019). Fetal Growth Restriction Alters Cerebellar Development in Fetal and Neonatal Sheep. Front. Physiol..

[50] Castillo-Melendez M., Yawno T., Allison B.J., Jenkin G., Wallace E.M., Miller S.L. (2015). Cerebrovascular adaptations to chronic hypoxia in the growth restricted lamb. Int. J. Dev. Neurosci..

[51] Castillo-Melendez M., Yawno T., Sutherland A., Jenkin G., Wallace E.M., Miller S.L. (2017). Effects of antenatal melatonin treatment on the cerebral vasculature in an ovine model of fetal growth restriction. Dev. Neurosci..

[52] Linder N., Haskin O., Levit O., Klinger G., Prince T., Naor N., Turner P., Karmazyn B., Sirota L. (2003). Risk factors for intraventricular hemorrhage in very low birth weight premature infants: A retrospective case-control study. Pediatrics.

[53] Khanafer-Larocque I., Soraisham A., Stritzke A., Al Awad E., Thomas S., Murthy P., Kamaluddeen M., Scott J.N., Mohammad K. (2019). Intraventricular Hemorrhage: Risk Factors and Association with Patent Ductus Arteriosus Treatment in Extremely Preterm Neonates. Front. Pediatr..

[54] Roberts J.C., Javed M.J., Hocker J.R., Wang H., Tarantino M.D. (2018). Risk factors associated with intraventricular hemorrhage in extremely premature neonates. Blood Coagul. Fibrinolysis..

[55] Khazardoost S., Ghotbizadeh F., Sahebdel B., Akbarian-Rad Z., Pahlavan Z. (2019). Predictors of Cranial Ultrasound Abnormalities in Intrauterine Growth-Restricted Fetuses Born between 28 and 34 Weeks of Gestation: A Prospective Cohort Study. Fetal Diagn. Ther..

[56] Marsoosi V., Bahadori F., Esfahani F., Ghasemi-Rad M. (2012). The role of Doppler indices in predicting intra ventricular hemorrhage and perinatal mortality in fetal growth restriction. Med. Ultrason..

[57] Bernstein I.M., Horbar J.D., Badger G.J., Ohlsson A., Golan A. (2000). Morbidity and mortality among very-low-birth-weight neonates with intrauterine growth restriction. The Vermont Oxford Network. Am. J. Obstet. Gynecol..

[58] Gilbert W.M., Danielsen B. (2003). Pregnancy outcomes associated with intrauterine growth restriction. Am. J. Obstet. Gynecol..

[59] Ortigosa Rocha C., Bittar R.E., Zugaib M. (2010). Neonatal outcomes of late-preterm birth associated or not with intrauterine growth restriction. Obstet. Gynecol. Int..

[60] Eixarch E., Meler E., Iraola A., Illa M., Crispi F., Hernandez-Andrade E., Gratacos E., Figueras F. (2008). Neurodevelopmental outcome in 2-year-old infants who were small-for-gestational age term fetuses with cerebral blood flow redistribution. Ultrasound Obstet. Gynecol..

[61] Oros D., Figueras F., Cruz-Martinez R., Padilla N., Meler E., Gratacos E., Cruz-Martinez R., Hernandez-Andrade E. (2010). Middle versus anterior cerebral artery Doppler for the prediction of perinatal outcome and neonatal neurobehavior in term small-for-gestational-age fetuses with normal umbilical artery Doppler. Ultrasound Obstet. Gynecol..

[62] Baschat A.A. (2014). Neurodevelopment after fetal growth restriction. Fetal Diagn. Ther..

[63] Lubrano C., Taricco E., Coco C., Di Domenico F., Mandò C., Cetin I. (2022). Perinatal and Neonatal Outcomes in Fetal Growth Restriction and Small for Gestational Age. J. Clin. Med..

[64] Mileusnić-Milenović R. (2021). Higher frequency of germinal matrix-intraventricular hemorrhage in moderate and late preterm and early term neonates with intrauterine growth restriction compared to healthy ones. Acta Clin. Croat..

[65] Sanz-Cortes M., Egaña-Ugrinovic G., Simoes R.V., Vazquez L., Bargallo N., Gratacos E. (2015). Association of brain metabolism with sulcation and corpus callosum development assessed by MRI in late-onset small fetuses. Am. J. Obstet. Gynecol..

[66] Simões R.V., Cruz-Lemini M., Bargalló N., Gratacós E., Sanz-Cortés M. (2015). Brain metabolite differences in one-year-old infants born small at term and association with neurodevelopmental outcome. Am. J. Obstet. Gynecol..

[67] Arcangeli T., Thilaganathan B., Hooper R., Khan K.S., Bhide A. (2012). Neurodevelopmental delay in small babies at term: A systematic review. Ultrasound Obstet. Gynecol..

[68] Ahn C., Lee J.S., Jeung E.B. (2013). 128 Expressions of mouse tight junction molecules in placenta—claudins and other paracellular transport molecules. Reprod. Fertil. Dev..

[69] Marzioni D., Banita M., Felici A., Paradinas F.J., Newlands E., De Nictolis M., Mühlhauser J., Castellucci M. (2001). Expression of ZO-1 and occludin in normal human placenta and in hydatidiform moles. Mol. Hum. Reprod..

[70] Leach L., Lammiman M.J., Babawale M.O., Hobson S.A., Bromilou B., Lovat S., Simmonds M.J. (2000). Molecular organization of tight and adherens junctions in the human placental vascular tree. Placenta.

[71] Guidoni P.B., Pasternak J.A., Hamonic G., MacPhee D.J., Harding J.C.S. (2021). Decreased tight junction protein intensity in the placenta of porcine reproductive and respiratory syndrome virus-2 infected fetuses. Placenta.

[72] Zhang Y., Zhao H.J., Xia X.R., Diao F.Y., Ma X., Wang J., Gao L., Liu J., Gao C., Cui Y.G. (2019). Hypoxia-induced and HIF1α-VEGF-mediated tight junction dysfunction in choriocarcinoma cells: Implications for preeclampsia. Clin. Chim. Acta..

[73] Liévano S., Alarcón L., Chávez-Munguía B., González-Mariscal L. (2006). Endothelia of term human placentae display diminished expression of tight junction proteins during preeclampsia. Cell Tissue Res..

[74] Gordijn S.J., Beune I.M., Thilaganathan B., Papageorghiou A., Baschat A.A., Baker P.N., Silver R.M., Wynia K., Gan-zevoort W. (2016). Consensus definition of fetal growth restriction: A Delphi procedure. Ultrasound Obstet. Gynecol..

[75] Hadlock F.P., Harrist R.B., Martinez-Poyer J. (1991). In utero analysis of fetal growth: A sonographic weight standard. Radiology.

[76] Hadlock F.P., Deter R.L., Harrist R.B., Park S.K. (1984). Estimating fetal age: Computer-assisted analysis of multiple fetal growth parameters. Radiology.

[77] Magann E.F., Sanderson M., Martin J.N., Chauhan S. (2000). The amniotic fluid index, single deepest poecket and two diameter poeket in normal pregnancy. Am. J. Obstet. Gynecol..

[78] Baschat A.A., Gembruch U. (2003). The cerebroplacental Doppler ratio revisited. Ultrasound Obstet. Gynecol..

[79] Sekizuka N., Hasegawa I., Takakuwa K., Tanaka K. (1997). Scoring of uterine artery flow velocity waveform in the assessment of fetal growth restriction and/or pregnancy induced hypertension. J. Matern. Fetal Investig..

[80] Gudmundsson S., Korszun P., Olofsson P., Dubiel M. (2003). New score indicating placental vascular resistance. Acta Obstet. Gynecol. Scand..

[81] Szczapa J., Wojsyk-Banaszak I., Dobrzańska A., Ryżko J. (2014). Wcześniactwo. Pediatria. Podręcznik do Lekarskiego Egzaminu Końcowego i Państwowego Egzaminu Specjalizacyjnego.

[82] Korones S.B. (1981). High Risk Newborn Infants. The Basis for Intensive Nursing Care.

[83] Lowry O.H., Rosebrough N.J., Farr A.L., Randall R.J. (1951). Protein measurement with the Folin phenol reagent. J. Biol. Chem..

[84] Papile L.A., Burstein J., Burstein R., Koffler H. (1978). Incidence and evolution of subependymal and intraventricular hemorrhage: A study of infants with birth weights less than 1500 gm. J. Pediatr..

[85] Baker L.L., Stevenson D.K., Enzmann D.R. (1988). End-stage periventricular leukomalacia: MR evaluation. Radiology.

[86] Barkovich A.J., Truwit C.L. (1990). Brain damage from perinatal asphyxia: Correlation of MR findings with gestational age. AJNR Am. J. Neuroradiol..

